# Evaluating the Prospects of Ti-Base Lattice Infiltrated with Biodegradable Zn–2%Fe Alloy as a Structural Material for Osseointegrated Implants—In Vitro Study

**DOI:** 10.3390/ma14164682

**Published:** 2021-08-19

**Authors:** Noa Gabay, Tomer Ron, Razi Vago, Amnon Shirizly, Eli Aghion

**Affiliations:** Department of Materials Engineering, Ben-Gurion University of the Negev, Beer Sheva 8410501, Israel; toron@post.bgu.ac.il (T.R.); rvago@bgu.ac.il (R.V.); a.shirizly@gmail.com (A.S.); egyon@bgu.ac.il (E.A.)

**Keywords:** osseointegration, additive manufacturing, SLM, lattice, Ti–6Al–4V, biodegradable Zinc, Zn–Fe, orthopedic, dental

## Abstract

The term “osseointegrated implants” mainly relates to structural systems that contain open spaces, which enable osteoblasts and connecting tissue to migrate during natural bone growth. Consequently, the coherency and bonding strength between the implant and natural bone can be significantly increased, for example in operations related to dental and orthopedic applications. The present study aims to evaluate the prospects of a Ti–6Al–4V lattice, produced by selective laser melting (SLM) and infiltrated with biodegradable Zn2%Fe alloy, as an OI–TiZn system implant in in vitro conditions. This combined material structure is designated by this study as an osseointegrated implant (OI–TiZn) system. The microstructure of the tested alloys was examined both optically and using scanning electron microscopy (SEM) and X-ray diffraction (XRD) analysis. The mechanical properties were assessed in terms of compression strength, as is commonly acceptable in cases of lattice-based structures. The corrosion performance was evaluated by immersion tests and electrochemical analysis in terms of potentiodynamic polarization and electrochemical impedance spectroscopy (EIS), all in simulated physiological environments in the form of phosphate buffered saline (PBS) solution. The cytotoxicity was evaluated in terms of indirect cell viability. The results obtained demonstrate the adequate performance of the OI–TiZn system as a non-cytotoxic structural material that can maintain its mechanical integrity under compression, while presenting acceptable corrosion rate degradation.

## 1. Introduction

The initial definition of osseointegration claimed by Albrektsson et al. [1] was “a direct contact between a loaded implant surface and bone” that can practically improve the mechanical bonding between the permanent implant and the bone [2,3]. Currently, the novelty of producing implants through additive manufacturing (AM) technologies directly from a computer model enable the production of complex implants [4,5,6,7] that can be designated for a specific patient. In particular, this also relates to the capability of producing cellular material in the form of a structural lattice that inherently incorporates open space volume [8]. The structural lattice can then be infiltrated with a biodegradable alloy that slowly dissolves in in vivo conditions. In parallel, bone tissue can grow within the open space generated by the disintegration of the biodegradable alloy. Consequently, this can stimulate osseointegration processes in terms of improving the mechanical bonding between the natural bone and the permanent implant. A relatively similar type of approach was carried out by Balog et al. [9] by producing a composite system using an extruded mixture of Ti and 12% biodegradable Mg powder. They found that the degradation of Mg post-implantation could be replaced by bone tissue that enhances osseointegration. Another example of this type of approach was introduced by Jiang et al. [10]. They demonstrated that a Ti–Mg system obtained by infiltrating pure biodegradable Mg into porous Ti matrix that was produced by applying controlled pressure on Ti wires within a cylindrical preform die.

The commonly preferred additive manufactured metal lattice for osseointegrated implants is Ti–6Al–4V alloy due to its outstanding mechanical properties and excellent biocompatibility [11,12,13,14]. Although Ti–6Al–4V alloy is widely used as a structural material for implants, sometimes it has the tendency to generate problems, such as stress shielding and cytotoxicity that is mainly attributed to the presence of vanadium. Hence, it should be pointed out that other Ti-base alloys are also being considered as of late, such as beta-type Ti–Nb–Zr–Mn alloys [15], Ti25Nb–13Ta–5Zr [16], and Ti–Al–Nb [17]. As for the infiltrated biodegradable metal, there are basically three common alternatives: Fe-base, Mg- base, and Zn-base alloys. Fe-based alloys tend to produce voluminous oxides that repel neighboring tissue, while Mg-based alloys suffer from accelerated corrosion rates that are associated with hydrogen gas evolution and an inherent risk of gas embolism [18,19,20,21,22]. In contrast, Zinc exhibited none of these disadvantageous characteristics [23,24,25,26,27,28]. In fact, Zn tends to stimulate bone growth [29] and act as an antibacterial material that is significantly effective in fighting infections [30,31,32]. However, research carried out by Guillory et al. [33] indicated that pure Zn may suffer from insufficient corrosion degradation rates in in vivo conditions that consequently can provoke fibrous encapsulation and limit the proper dissolution of the implant. To prevent this phenomenon, Fe and other alloying elements were added to pure Zinc in order to accelerate the corrosion rate by a micro-galvanic effect [34]. The present study aims to evaluate the prospects of a Ti–6Al–4V lattice infiltrated with biodegradable Zn–2%Fe alloy as a structural material system for osseointegrated implants in in vitro conditions.

## 2. Materials and Methods

### 2.1. Preparation of Osseointegrated Implants (OI–TiZn) System

The OI–TiZn system was made from a permanent Ti–6Al–4V lattice produced by AM, which was subsequently infiltrated by a biodegradable Zn–2%Fe alloy. The Ti–6Al–4V lattice was fabricated by an SLM process using an EOS-EOSINT M280 facility equipped with a 200 W Nd-YAG laser. The particle size of the Ti–6Al–4V powder was between 20–45 µm. The laser beam diameter was 60 µm, the scanning velocity was 7000 mm/s, the hatch spacing was 0.2 mm, and the building layer thickness was 30 µm. The SLM scanning direction rotation was 45 degrees, and the lattice unit cell was in the form of a cubic structure with a 2.5 mm cell parameter and a rod diameter of 1 mm. All the AM lattices were manufactured in the Z-direction and in accordance with ASTM standard 52921-13. The selection of Zn–2%Fe as the biodegradable alloy mainly relates to the relative advantage of this alloy composition in terms of corrosion degradation [18,34]. This selection aims to avoid the danger of insufficient corrosion degradation rates, which can promote fibrous encapsulation and limit the dissolution of the infiltrated alloy [33]. The Zn–2%Fe was produced by gravity casting using pure Zn ingots (99.99%) and pure Fe (99%) powder with a mean grain size of 44 microns. The casting process of Zn–2%Fe was carried out in a graphite crucible and included the following stages: (i) re-melting of pure Zn at 750 °C, and (ii) adding Fe powder with subsequent stirring every 30 min for 3 h [18]. Prior to the infiltration of the biodegradable Zn alloy, the Ti alloy lattice was pre-heated at 400 °C for 1 h. The infiltration process was carried out by immersing the Ti alloy lattice in molten Zn alloy at a temperature above 700 °C within a steel die. In order to secure the proper filling of the Ti-base lattice, the infiltration process was accompanied by intensive shaking.

### 2.2. Microstructure Examinations

The microstructure of the tested materials was examined using JSM-5600 (Tokyo, Japan) scanning electron microscopy (SEM) integrated with an energy dispersive X-ray spectroscopy (EDS) detector (Thermo Fisher Scientific, Waltham, MA, USA) for localized chemical composition analysis with a spot size of 1 μm [35]. Phase identification was carried out by X-ray diffraction (XRD) analysis, using an X-ray diffractometer (RIGAKU-2100H (Tokyo, Japan) with Cu–Kα. Diffraction patterns were generated in the range of 20°–90° at 40 kV, 30 mA, and a scanning rate of 0.02°/min. The additively manufactured Ti6Al4V lattice and biodegradable Zn–2%Fe alloy were separately tested as monolithic bulk materials. In order to examine the phases composing the interface between the Ti lattice and the biodegradable Zn-based alloy, the OI–TiZn system was tested in the form of powder subsequent to the removal of intact Ti-base lattice rods. Prior to the XRD analysis, the final powder was ground to obtain a fine grain size of about 100 μm.

### 2.3. Compression Strength

The mechanical properties of the Ti-lattice and the OI–TiZn system were evaluated in terms of compressive strength, as is commonly acceptable in analyzing three dimensional lattices [10,36]. This was carried out using a Hounsfield H25 KT testing facility with a crosshead-speed of 0.5 mm/min. The test specimens had a cylindrical shape with dimensions of a 10 mm diameter and a 20 mm length, and were obtained by an electro-erosion cutting machine (ARTA 123Pro EDM).

### 2.4. Immersion Test

The corrosion resistance of the OI–TiZn system and of the biodegradable Zn–2%Fe alloy in equilibrium conditions were examined in terms of immersion tests for 14 days. The testing solution was phosphate buffered saline (PBS) and the temperature was 37 °C, respectively, to simulate typical physiological environmental conditions in accordance with the ASTM ID: G31-72 standard. The specimens for the immersion tests were in the form of cylindrical rods with a 10 mm diameter and an 8 mm length. The specimens were produced by an ARTA 123Pro EDM electro-erosion cutting machine.

The corrosion rate was calculated according to the following equation:

(1) $$CR=\frac{W}{A\cdot T\cdot D}$$

where *W* is the weight loss, *A* is the exposed area, *T* is the duration time, and *D* is the density of the specimen.

### 2.5. Electrochemical Behavior

The electrochemical behavior of the tested materials was evaluated using a Bio-Logic SP-200 potentiostat equipped with EC-Lab software V11.18. This was also carried out in the PBS solution using a three-electrode cell that included a working electrode with an exposed area of 1 cm^2^, a saturated calomel electrode (SCE) as a reference electrode, and a platinum counter electrode. The scanning rate of potentiodynamic polarization analysis was 1 mV/s. The electrochemical impedance spectroscopy (EIS) analysis was carried out within the range of 10 kHz–100 MHz at 10 mV amplitude. Prior to all electrochemical analysis, the samples were polished to 1200 grit. The corrosion rate was evaluated by Tafel extrapolation [37].

### 2.6. Cytotoxicity Testing

Cytotoxicity testing was carried out by evaluating the effect of indirect extract on cell metabolic activity according to the ISO 10993-5/12 standard. The cytotoxicity analysis was conducted using Mus musculus (mouse) 4T1 cells, due to their relatively increased sensitivity to toxic effects. In fact, those cells are significantly more active than primary cells, and consequently more sensitive to toxic conditions. Prior to the cytotoxicity tests, Ti–6Al–4V samples produced by an SLM process and OI–TiZn samples—both having a diameter of 7.9 mm and a length of 2 mm—underwent the following treatments: (i) polished up to 4000 grit, (ii) cleaned in ethanol for 10 min, (iii) cleaned in acetone for 5 min, and (iv) sterilized in UV light for 1 h on each side. The required extract was obtained by immersing the related specimens in Dulbecco Modified Eagle’s Medium (DMEM) solution with 4.5 g/L D-Glucose, 10% Fetal Bovine Serum (FBS), 4 mM L-Glutamine, 1 mM Sodium Pyruvate (SP), and 1% Penicillin Streptomycin Neomycin (PSN) antibiotic mixture under a 5% CO_2_ humidified atmosphere for 24 h at 37 °C. The surface area to volume extraction ratio was 1.25 ${\mathrm{cm}}^{2}$/mL. The extracts were then collected and filtered using a PVDF membrane of 0.45 μm. The 4T1 cells were seeded in 96-well tissue culture plates, in a concentration of 5000 cells per well, and incubated for 24 h to allow for proper rehabilitation. The cells’ medium was then replaced by 100 μL of the following extracts (4 wells of each group): Ti–6Al–4V as the control group, and OI–TiZn extracts, diluted to 1:10 (10%) to correspond to the biological ability to naturally dilute and rid excesses of Zn [38,39]. As additional positive and negative control groups, 4 wells’ mediums were replaced with 100 μL of only DMEM, and 4 wells’ mediums were replaced with 100 μL of 90% DMEM and 10% DMSO, respectively. Two identical 96-well plates were incubated for 24 h and 48 h. The cell viability was assessed using a Cell Proliferation Kit (XTT, Biological Industry, Beit Haemek, Israel). According to this process, each sample (in addition to 3 blank wells as reference) was incubated for 2 h in 100 μL DMEM, 50 μL reagent, and 1 μL activator. The color transformation was measured spectrophotometrically at 490 nm using a microplate reader (SYNERGY-Mx, BioTek, Winooski, VT, USA). The obtained values were compared with the control blank samples in order to assess the cell viability. The general appearance of each sample was visually examined using a CoolLED pE-2 collimator fitted to an inverted phase-contrast microscope (Eclipse Ti, Nikon, Tokyo, Japan) equipped with a digital camera (DS-Qi1Mc, Nikon, Tokyo, Japan). The tests were repeated twice independently to validate the obtained results.

## 3. Results

### 3.1. Preparation of OI–TiZn Samples and Microstructure Examinations

**Figure 1:**
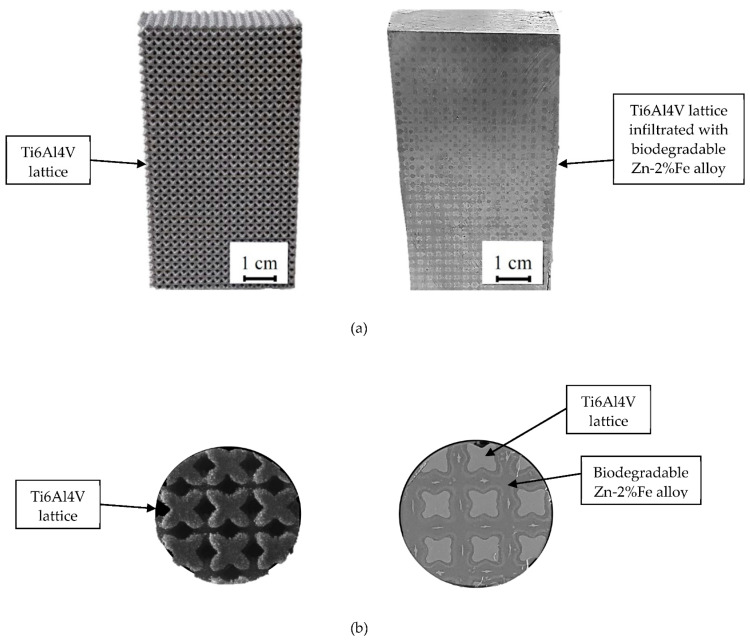
(**a**,**b**) General and close-up views of Ti-base lattice and infiltrated lattice (OI–TiZn system), respectively.

The general appearance of the Ti–6Al–4V lattice obtained by the SLM process is shown in Figure 1a. This illustrates the symmetric cubic structure of the lattice, as well as the locations of open spaces marked by the darker areas. A close-up view of the Ti-base lattice infiltrated with biodegradable Zn–2%Fe alloy is shown in Figure 1b. This clearly reveals that the infiltrated alloy was able to both penetrate and fill the open spaces within the lattice with good matching to the internal titanium structure.

**Figure 2:**
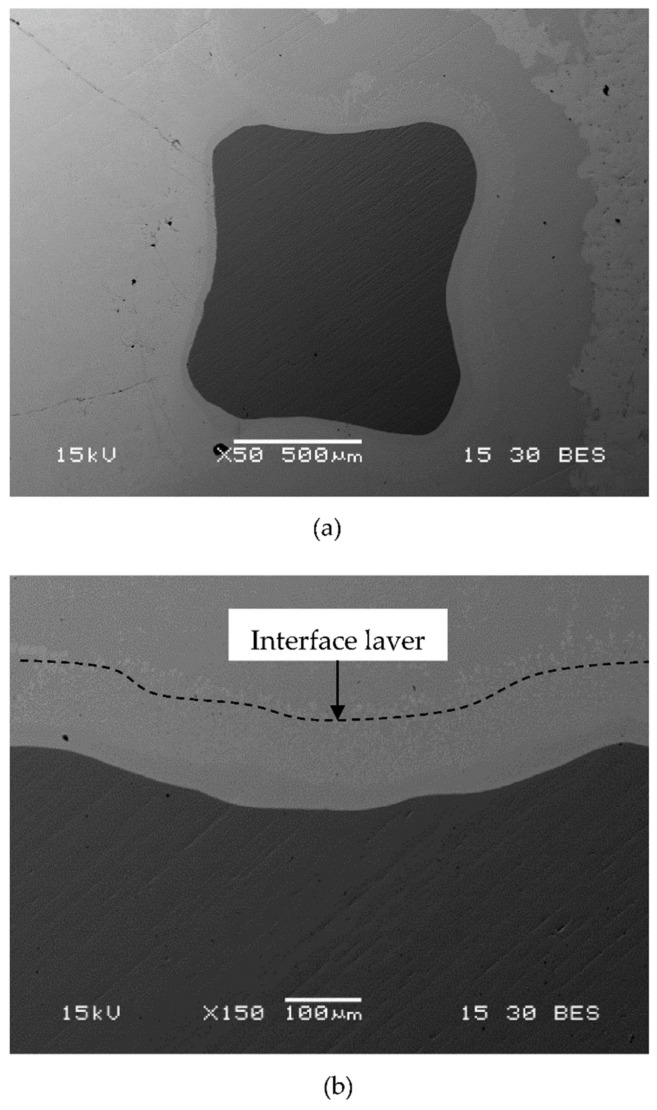
General appearance of the boundary between the Ti–6Al–4V lattice and the infiltrated Zn–2%Fe alloy: (**a**) close-up view and (**b**) magnified view of the interface layer between the two alloys.

A close-up view of the boundary between the Ti-base lattice and the infiltrated Zn–2%Fe alloy, as obtained by SEM back scatter electron (BSE) analysis, is shown in Figure 2a. A magnified view of that boundary clearly discloses the presence of an interface layer with a width of about 130 µm attached to the Ti-base rods, as shown in Figure 2b.

**Figure 3:**
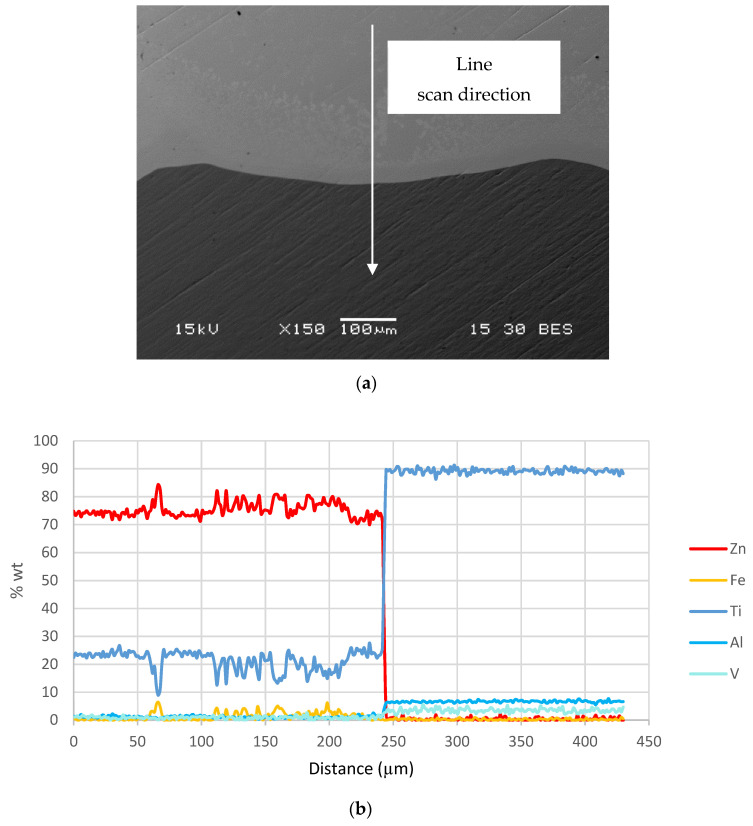
Chemical composition of the interface layer between the Ti-base lattice and infiltrated Zn–2%Fe alloy—(**a**) line scan direction and (**b**) chemical composition obtained by EDS analysis.

**Figure 4:**
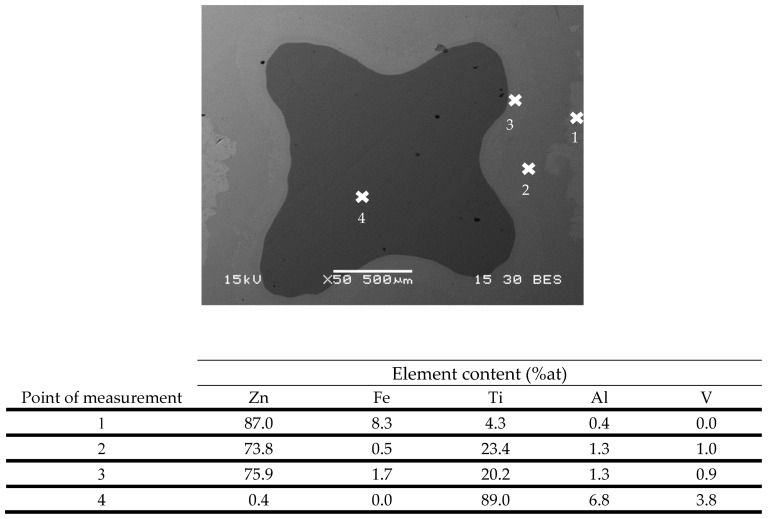
Spot chemical composition measurements along the border obtained by EDS analysis with an error of 2%at.

In order to evaluate the characteristics of the interface layer between the Ti–base lattice rods and the infiltrated Zn–2%Fe alloy, the chemical composition of this layer was analyzed by energy-dispersive spectroscopy (EDS), as shown in Figure 3. This has indicated that a relatively large amount of Ti was present in this layer, along with a large amount of Zn. According to the spot chemical analysis shown in Figure 4 (points 2 and 3), the amount of Ti in this layer was within the range of 20.2–23.4%, along with 0.5–1.7% Fe. In parallel, the content of other alloying elements composing the Ti-base lattice (Al and V) within this layer were relatively minor compared to their original content in the Ti–6Al–4V alloy.

**Figure 5:**
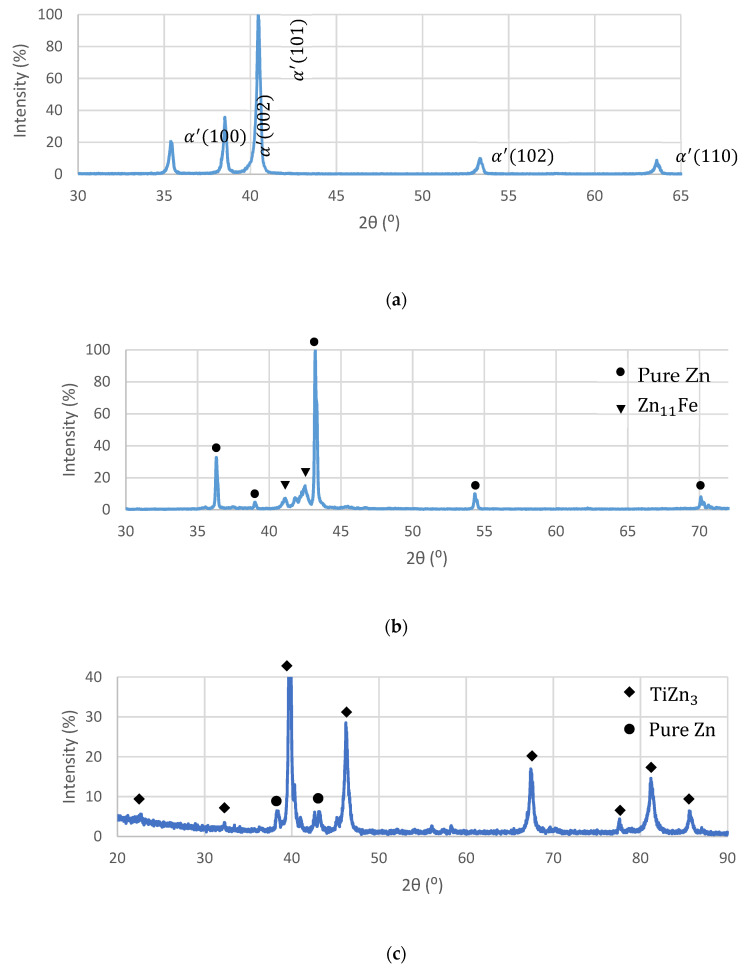
XRD analysis of (**a**) Ti–6Al–4V lattice, (**b**) Zn–2%Fe alloy, and (**c**) mixed powder of OI–TiZn system without the intact Ti-base lattice rods.

Further investigation of the interface layer was carried out by an XRD analysis. Figure 5a reveals the phase characteristics of the Ti-base lattice that was mainly composed of martensitic phase (α’) due to rapid solidification conditions, as expected from Ti–6Al–4V alloy produced by an SLM process. The XRD analysis of the biodegradable Zn–2%Fe alloy indicated the presence of pure Zn matrix and a secondary phase in the form of Zn_11_Fe, as shown in Figure 5b. The diffraction analysis of a mixed powder of the OI–TiZn system without the intact Ti-base lattice rods is shown in Figure 5c. This reveals the presence of pure Zn, along with a dominant secondary phase in the form of TiZn_3_. According to the ternary phase diagram (Fe–Ti–Zn) presented by Raghavan [40], the TiZn_3_ phase can be obtained in concentrations of over 95%wt Zn, 10–25%wt Ti, and 0–3% Fe. This comes in line with the spot chemical composition analysis at the interface layer, as shown in Figure 4, where the Ti content was 15.8–18.4%wt and the Fe content was 0.47–1.58%.

### 3.2. Mechanical Properties in Terms of Compression Strength

**Figure 6:**
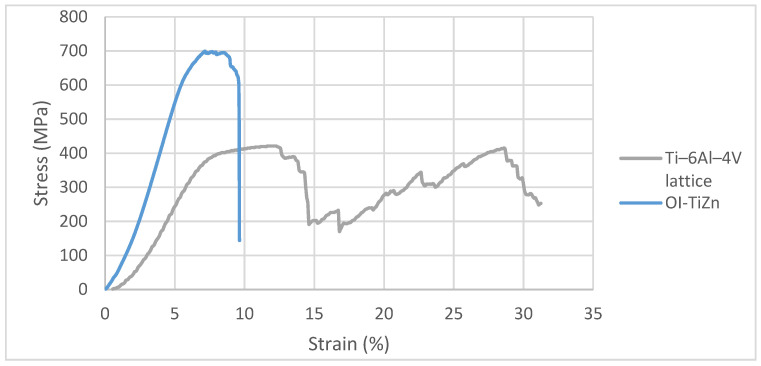
Typical compressive stress-strain curves of Ti–6Al–4V lattice and OI–TiZn system.

The mechanical properties of the Ti-base lattice and the OI–TiZn system along the XZ plane were evaluated in terms of compression tests. Typical stress-strain curves are shown in Figure 6, along with the statistical deviations of the mechanical properties as presented in Table 1. The nominal cross section area of the lattice was calculated using the following equation:

(2) $$A_{L}=V_{L}/h_{L}$$

where $A_{L}$ is the cross-section area of the lattice, $h_{L}$ is the height of the sample, and $V_{L}$ is the cylindrical volume of the specimen obtained by the following relation:

(3) $$V_{L}=W_{L}/\rho$$

where $W_{L}$ is the weight of the specimen, and ρ is the density, taken as 4.4 g/${\mathrm{cm}}^{3}$ [41].

**Figure 7:**
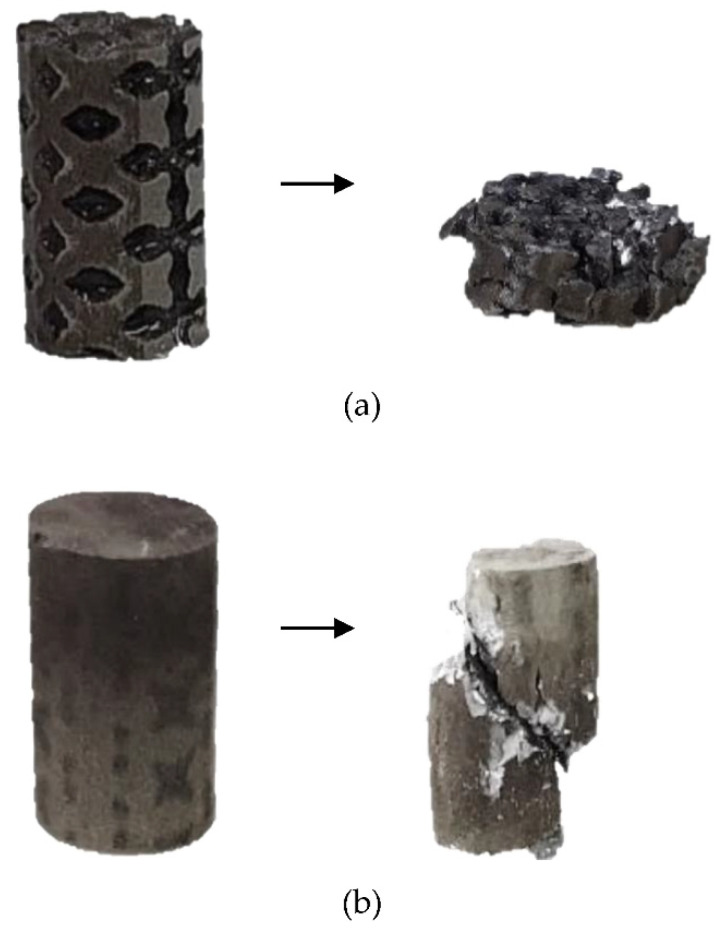
Typical mode of failure obtained under compression stress of (**a**) Ti–6Al–4V lattice, (**b**) OI–TiZn system.

The obtained results showed a repeated loss and recovery of strength by the Ti-alloy lattice that can be related to the collapsing of cells along XZ planes, as indicated by Maskery et al. [42]. In comparison, the stable strength performance of the OI–TiZn system with a naturally higher ultimate compressive strength (UCS) and yield strength compared to the Ti–base lattice is in line with the basic observations of Jiang et al. [10]. The failure mode of the Ti-base lattice shown in Figure 7a displays a layer-by-layer crushing due to shear deformation. Contrarily, the mode of failure of the OI–TiZn system (Figure 7b) was dominated by the infiltrated Zn–2%Fe alloy and resulted in pure shear fracture, while maintaining the important mechanical integrity of the OI–TiZn system. According to Jiang et al. [36], this type of fracture can be related to the critical resolved shear stress developed along the internal planes of the OI–TiZn system.

### 3.3. Corrosion Resistance and Electrochemical Behavior

**Figure 8:**
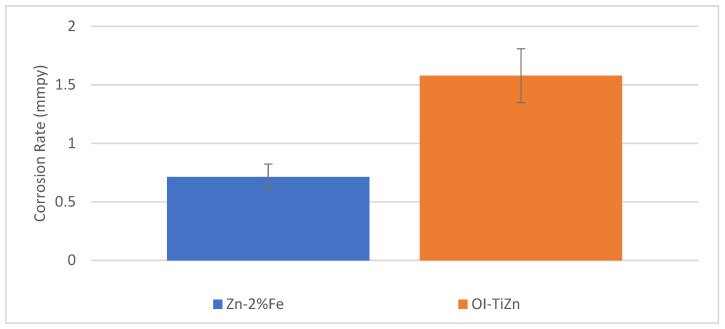
Corrosion rate measurements obtained after immersion tests of Zn–2%Fe alloy and OI–TiZn system in PBS solution at 37 °C for 14 days.

**Figure 9:**
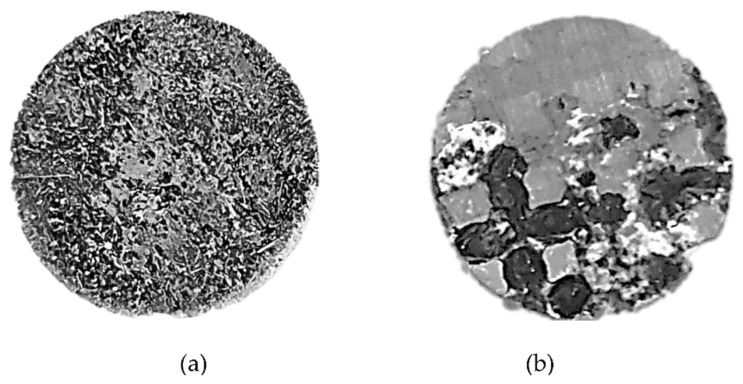
General appearance of corrosion attack obtained after 14 days of immersion test: (**a**) Zn–2%Fe alloy and (**b**) OI–TiZn system.

**Figure 10:**
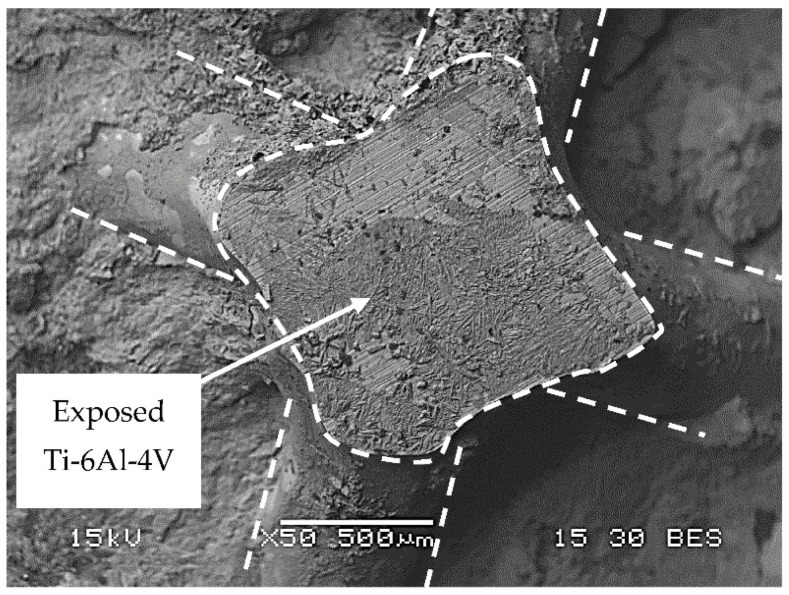
A magnified view of the galvanic corrosion attack in the vicinity of the Ti-base lattice after immersion test for 14 days.

The corrosion performance of the biodegradable Zn–2%Fe alloy and the OI–TiZn system under an immersion test in the PBS solution at 37 °C for 14 days is shown in Figure 8. This reveals that the corrosion rate of the OI–TiZn system was significantly higher, apparently due to the galvanic effect created between the Ti-base lattice and the infiltrated Zn–2%Fe alloy. This assumption was clearly supported by the general corrosion attack views shown in Figure 9, which demonstrate the increased corrosion attack at the boundary between the Ti-base rods and infiltrated alloy. A magnified view of this galvanic corrosion is shown in Figure 10. This noticeably reveals the significant degradation of the infiltrated Zn–2%Fe alloy, and the vicinity of the Ti-base lattice that was kept nearly intact in parallel.

**Figure 11:**
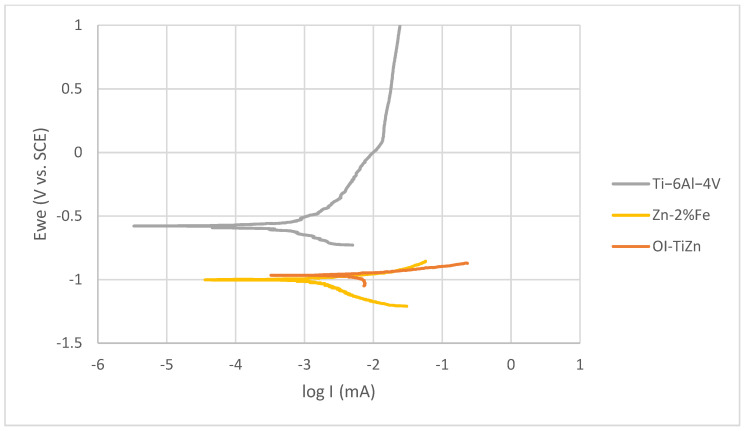
Potentiodynamic polarization analysis of Ti–6Al–4V lattice, Zn–2%Fe alloy, and OI–TiZn system in PBS solution.

An electrochemical analysis in terms of potentiodynamic polarization of Ti–6Al–4V alloy, biodegradable Zn–2%Fe alloy, and the OI–TiZn system is shown in Figure 11. This indicates that the polarization curve of the OI–TiZn system was shifted to higher current densities, indicating an increased corrosion rate [43]. This was supported by Tafel extrapolation (Table 2), which clearly revealed that the corrosion rate of the OI–TiZn system was higher by about three orders of magnitude as compared to the Ti-base lattice, and by less than two orders of magnitude as compared to the biodegradable Zn–2%Fe alloy. This electrochemical outcome clearly demonstrates the dominant effect of the infiltrated Zn–2%Fe alloy on corrosion tendencies of the OI–TiZn system. In addition, these results comply with the results obtained by the immersion tests, and the assumptions related to the substantial effects of galvanic corrosion.

**Figure 12:**
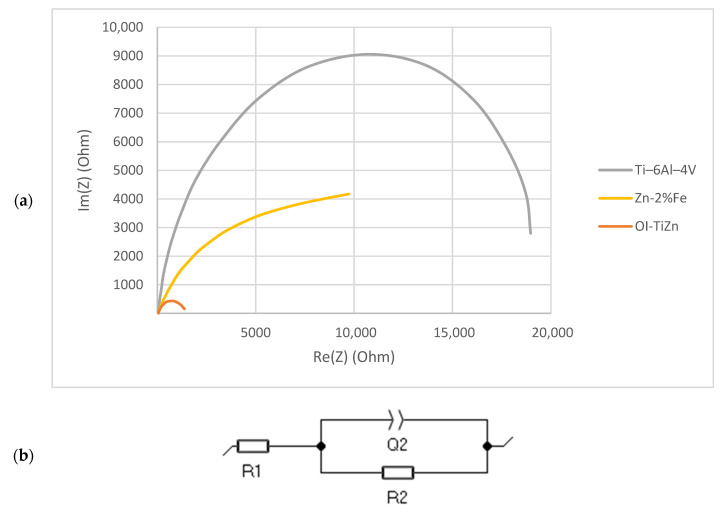
(**a**) Nyquist plots of Ti-base lattice, Zn–2%Fe alloy, and OI–TiZn system; (**b**) fitted electrical equivalent circuit (EEC).

A further investigation of the electrochemical behavior of the tested materials was carried out using EIS in terms of a Nyquist plot, as shown in Figure 12a. This clearly shows a significant reduction in the radii of curvature of the OI–TiZn system as compared to both Ti-base lattice and Zn–2%Fe alloy. According to Kafri et al. [18], the reduced radius of curvature is an indication of a reduced corrosion resistance. The electrical equivalent circuit fitted to the Nyquist plots is shown in Figure 12b, where R1 represents the solution resistance, R2 the specimen’s resistance, and Q2 the capacitor created by the double layer. The values derived from the plots in correlation with the electrical circuit are shown in Table 3. This indicates that the resistance R2 of the OI–TiZn system was reduced by one order of magnitude, as compared to that of the Ti-base lattice alloy and the Zn–2%Fe alloy. Altogether, the results of the EIS are in accord with the outcome obtained by the potentiodynamic polarization analysis and the immersion test.

### 3.4. Cytotoxicity Analysis

**Figure 13:**
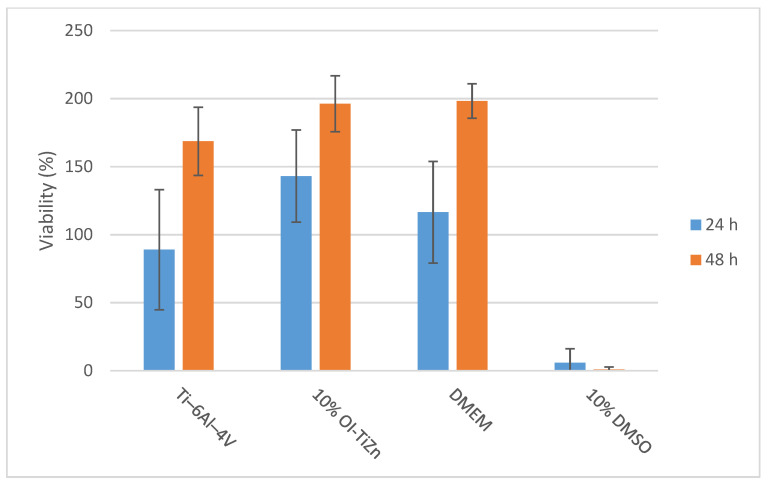
Viability analysis of 4T1 cells in terms of their metabolic activity after incubation of 24 h and 48 h in extracts of the tested materials.

**Figure 14:**
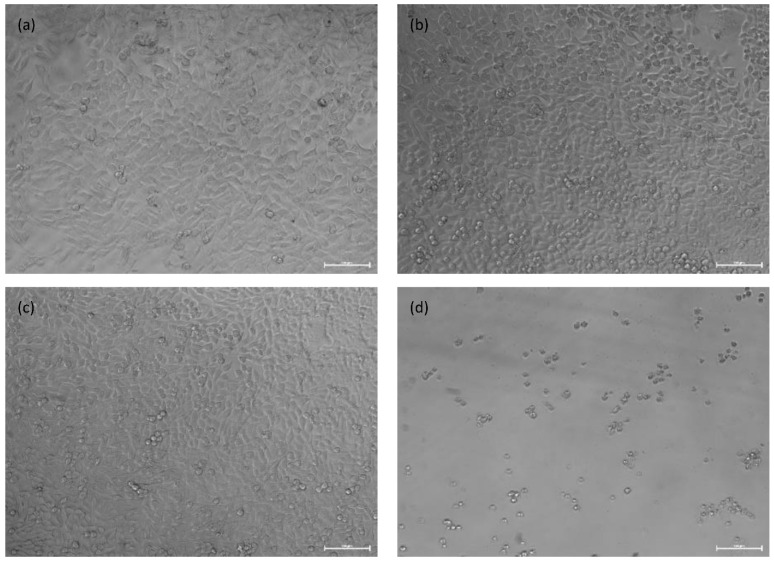
General appearance of 4T1 cells after 48 h incubation in extracts of: (**a**) Ti–6Al–4V alloy, (**b**) 10% OI–TiZn, (**c**) DMEM, and (**d**) 10% DMSO.

An indirect cytotoxicity analysis of the OI–TiZn system and the Ti–6Al–4V reference alloy that has excellent biocompatible properties [44] was carried out following incubation periods of 24 h and 48 h, as shown in Figure 13, in terms of cell viability. In the case of the OI–TiZn system, the dilution rate was 10% in order to rectify the natural buffering gap that exists between the in vivo and in vitro conditions [38,45]. The obtained results clearly indicate that both the Ti–6Al–4V alloy and the OI–TiZn system can be considered to be non-cytotoxic substrates for 4T1 cells due to a cell viability above 70%. These quantitative results comply with ISO-10993-5 (tests for in-vitro cytotoxicity) [46], which indicates that a viability reduction of up to 30% is not considered to be cytotoxic. The general appearance of the 4T1 cells after 48 h, as shown in Figure 14, complies with the quantitative analysis. The cells incubated in extracts of 10% OI–TiZn, Ti–6Al–4V alloy, and DMEM only, look normal and healthy.

## 4. Discussion

The attractiveness of an AM lattice infiltrated with a biodegradable alloy as a potential structural material for implants mainly relates to its capability to stimulate osseointegration bonding [2,3]. Furthermore, AM technologies enable the production of complex implants that can address, for example, special personal needs in dental and orthopedic operations. In the case of the OI–TiZn system, the potential osseointegration bonding can be implemented by the dissolution of the infiltrated biodegradable Zn–2%Fe alloy and, in parallel, the formation of bone tissue within the open space of the permanent Ti-base lattice implant. Other inherent advantages of OI–TiZn systems relate to the capability of Zinc to stimulate bone growth [29] and to act as an antibacterial substance that can prevent infections post-implantation.

The results obtained by this study in in vitro conditions clearly demonstrate the adequate bonding created between the biodegradable Zn–2%Fe alloy and the Ti-base lattice post-infiltration. This was naturally manifested by a significant increase in strength of the OI–TiZn system as compared to the monolithic lattice structure in terms of UCS—699 vs. 430 MPa, and yield strength—606 vs. 396 MPa. In addition, the fact that the mechanical integrity of the OI–TiZn was maintained following the compression test failure can also serve as an indication of the satisfactory bonding between the degradable alloy and the Ti lattice. The adequate matching between the infiltrated Zn–2%Fe alloy and the Ti-base lattice was also visible through the microstructure analysis. It is believed that the interface layer, with a width of about 130 µm that includes TiZn_3_ precipitates, is created by mutual diffusion between the infiltrated alloy and the Ti-base lattice. Consequently, this can contribute to the improved bonding between those two materials.

The corrosion rate of the OI–TiZn system in terms of the immersion test, potentiodynamic polarization, and EIS analysis in a simulated physiological solution was basically similar and within the range of about 0.15–1.6 mmpy. Although this was relatively increased as compared to the corrosion rate of the biodegradable Zn–2%Fe alloy (0.033 ± 0.75 mmpy) due to microgalvanic effects, this can be basically tolerated in in vivo conditions. In fact, according to Guillory et al. [33], arterial implants produced from pure Zn with corrosion rates of about 0.02–0.05 mmpy may provoke fibrous encapsulation that can isolate the implant and arrest the biodegradation process [47,48]. As a result, the relatively increased corrosion rate of the OI–TiZn system can be considered to be an inherent advantage that may prevent the potential danger of encapsulation processes in in vivo conditions. Furthermore, the prospects of the OI–TiZn system as an adequate implant material were also supported by the indirect cytotoxicity evaluation analysis in terms of cell viability. This clearly indicated that the OI–TiZn system can be related as a non-cytotoxic substance to relatively sensitive 4T1 cells. The average cells’ viability on the OI–TiZn system after 24 h and 48 h of incubation was 140% and 190%, respectively, which is well above the required cell viability of 70%. In addition, it was evident that the general appearance of the cells post-incubation was quite normal and healthy.

## 5. Conclusions

The in vitro results obtained by this study clearly demonstrate the prospects of an OI–TiZn system as an adequate structural material for implants that can potentially improve osseointegration bonding. This was illustrated by indirect cytotoxicity analysis in terms of cell viability, which showed that an OI–TiZn system can be related as a non-cytotoxic substance to relatively sensitive 4T1 cells. Although the corrosion rate of the OI–TiZn system in a simulated physiological solution was slightly increased due to microgalvanic effects, the obtained degradation rate can be considered to be quite tolerable. The adequate bonding obtained between the Ti-base lattice and the infiltrated Zn–2%Fe alloy was able to maintain the mechanical integrity of the OI–TiZn system under compression test conditions. This study should be followed by in vivo experiments that will demonstrate the capability of an OI–TiZn system to act as the structural material to enhance osseointegration.

## Figures and Tables

**Table 1:** Statistical deviations of the mechanical properties under compressive loading of Ti-base lattice and OI–TiZn system.

	Ultimate Compressive Strength (UCS) (MPa)	Yield Point (MPa)
Ti–6Al–4V lattice	429.9 ± 6.3	397.9 ± 2.6
OI–TiZn system	699 ± 120	606 ± 53

**Table 2:** The results obtained by Tafel extrapolation including statistical deviations.

	Ti–6Al–4V	Zn–2%Fe	OI–TiZn
$E_{\mathrm{corr}}\left(\bar{V}\right)\;\mathrm{vs}.\;\mathrm{SCE}$	−0.52 ± 0.06	−1.03 ± 0.05	−0.965 ± 0.001
$I_{\mathrm{corr}}\left(\mu A\right)$	0.80 ± 0.27	2.22 ± 2.05	9.95 ± 0.26
Corrosion Rate (mmpy)	0.005 ± 0.002	0.033 ± 0.031	0.149 ± 0.004

**Table 3:** The electrical equivalent circuit parameters fitted from the Nyquist plots.

	Ti–6Al–4V	Zn–2%Fe	OI–TiZn
R1 (Ω)	48.2 ± 2.7	49.0 ± 1.0	75.9 ± 24.1
R2 (Ω)	21,106 ± 4276	10,031 ± 1963	1182 ± 167
Q2 (μF)	47 ± 4	137 ± 65	85 ± 7

## Data Availability

Data Sharing Not Applicable.
